# Evaluation of the expression of LC3-II and BECLIN1 genes of autophagy pathway in patients with hematological malignancies 

**DOI:** 10.22088/cjim.14.4.694

**Published:** 2023

**Authors:** Hossein Ayatollahi, Samaneh Boroumand-Noughabi, Gordon Ferns, Maryam Sheikhi, Payam Siyadat, Mehrdad Rostami, Zahra Khoshnegah

**Affiliations:** 1 Department of Hematology and Blood Banking, Faculty of Medicine, Mashhad University of Medical Sciences, Mashhad, Iran; 2 Cancer Molecular Pathology Research Center, Mashhad University of Medical Sciences, Mashhad, Iran.; 3 Department of Medical Education, Brighton and Sussex Medical School, Brighton, UK; 4 Blood Transfusion Research Center, High Institute for Research and Education in Transfusion Medicine, Tehran, Iran; 5 Department of Hematology and Blood Banking, Faculty of Medicine, Gonabad University of Medical Sciences, Gonabad, Iran

**Keywords:** Autophagy, Hematological malignancy, *LC3*, *BECLIN1*, *AML*, *ALL*, *CML*

## Abstract

**Background:**

Autophagy is a pathway for the degradation of cytoplasmic components, which plays an essential role in various cellular and physiological processes, including cell renewal and survival, and immune responses. While recent studies have shown that they can play a role in cancer treatment, the precise mechanisms of autophagy in leukemogenesis are not fully understood. We have assessed the expression levels of LC3 and BECLIN1 as two crucial autophagy mediators in patients with leukemia.

**Methods:**

This cross-sectional study was performed on bone marrow or peripheral blood samples of 61 leukemia patients (24 AML, 20 ALL, and 17 CML) and compared to 18 healthy controls. Real-time PCR was used to quantitate gene expression. SPSS statistics 16.0 and Graph Pad Prism 8.4.2 software were applied for statistical analysis.

**Results:**

While BECLIN1 expression was significantly lower in AML, ALL, and CML patients as compared to the control group (p < 0.05), LC3 showed significantly different expression only in the AML patients (P= 0.03). There was no significant correlation between the expression levels of BECLIN1 with LC3 (p> 0.05). Whilst the AML LC3^high^ group had a significantly lower lymphocyte count (P= 0.023), the AML BECLIN1^low^ group had a significantly higher MPV levels (P= 0.044). Furthermore, ALL LC3^high^ group indicated a significantly lower HCT count (P= 0.017).

**Conclusion:**

Significant changes in the expression levels of BECLINI and LC3 in hematologic malignancies may indicate a possible role for autophagy in their pathogenesis. However, further studies are warranted to confirm these findings.

Autophagy is a pathway for the degradation of cytoplasmic components, which plays an essential role in various cellular and physiological processes, including cell renewal and survival, and immune responses (1, 2). Several reports indicate that autophagy may play a role in tumor formation, metastasis, protection against apoptosis, and support of cell survival. In addition, autophagy may lead to therapy resistance in tumor cells (3, 4). Some studies suggest that autophagy mechanisms that occur in the progression of leukemia, vary depending on the type and stage of leukemia, and also the type of oncogenes in tumor cell (5). Leukemia is a heterogeneous malignant disease of bone marrow hematopoietic stem cells. The most common types of leukemia include acute myeloid leukemia (AML), acute lymphoblastic leukemia (ALL), chronic myeloid leukemia (CML), and chronic lymphocytic leukemia (CLL) (6). Despite the proposed roles of autophagy as a central regulator of cellular metabolism, only a few studies focused on its role in different types of leukemia. Autophagy may balance quiescence, self-renewal, and differentiation of hematopoietic stem cells (HSCs) in a specialized nourishing niche in the bone marrow.

In this regard, animal studies have shown that conditional suppression of autophagic components disrupts the self-renewal of HSCs and thus significantly reduces the number of HSCs and progenitors of multiple lineages (7, 8). Several genes, including BECLIN1 and LC3, play a crucial function in mammalian autophagy (9, 10). Autophagy was first reported to be associated with the human breast carcinoma in 1999, when BECLIN1, an essential autophagy gene, was suggested to have a tumor-suppressive function with decreased expression (11, 12).Studies have shown BECLIN1 deletion as a significant autophagy factor in some human cancers (13). Also, it has been shown in various cancers, such as stomach cancer (14), colorectal cancer (15), and breast cancer (16), to be associated with the prognostic status of the disease. Microtubule-associated protein 1A/1B light chain 3 (*LC3*), the homolog of yeast *Atg8*, is crucial to forming autophagosomes in mammalian cells (17). According to previous findings, expression of LC3B can be used for monitoring of autophagy (18). In this study, we evaluated the expression levels of the *LC3* and *BECLIN1* as the markers of autophagy in patients with ALL, AML, and CML.

## Methods

**Study design and participants: **This cross-sectional study was undertaken in the Molecular Pathology Cancer Research Center, Mashhad University of Medical Sciences, Mashhad, Iran, from March to October 2021. The study was performed using bone marrow or peripheral blood samples collected in EDTA containing tubes of 61 (24 AML, 20 ALL and 17 CML) patients with leukemia, referred to the Hematology Unit of Ghaem University Hospital. The study was done on the same samples which were used for diagnostic purposes. In addition, 18 healthy controls (9 females, 9 males) were included. All patients’ clinical and molecular data were also collected from their archived medical records. This study was approved by the Research Ethics Committee of Mashhad University of Medical Sciences (IR.MUMS.MEDICAL.REC.1400.454).

**Real-time PCR: **The purity of extracted RNA was assessed using the Nano Drop 2000 Spectrophotometer (Thermo Scientific, USA). Then, Real-Time quantitative RT-PCR (RQ-PCR) reactions were performed by The Applied Bio-system Step One Plus Real-Time PCR Systems (Applied Bio-systems). The relative expression of BECLIN1 and LC3 genes in the same samples was evaluated by 2 ^-ΔΔCt^ method using *GAPDH* gene as the housekeeping gene. Primers for LC3 and BECLIN1 genes and the GAPDH gene (as an internal control) were designed by PubMed blast software. Table 1 shows the primer sequences used for each gene. Each qRT-PCR reaction contained 5 μL of master mix (SYBR Green), 2 μL of cDNA, 0.15 μL of each primer and probe, and DEPC water to reach a final volume of 10.5 μL. 40 cycles of 95°C for 5 min, denaturation at 95°C for 30s followed by extension at 60°C for 1.5 min were applied. All samples were analyzed in duplicate.

**Statistical analysis: **The minimum sample size for each group was calculated according to the Miracco C et al.’s study (19). The Shapiro- Wilk test was used to evaluate whether the data were normally distributed. Based on the distribution of the *LC3* and *BECLIN1* expression, non-parametric or parametric tests were used to compare the expression levels of LC3 and BECN1 between patients and controls. In addition, Pearson’s chi-squared test was used to measure the linear correlation between the two genes. We used fold change expression to assess the changes in expression of these genes. Fold change values of ≥2, between 0.5 to 2, and <0.5 were regarded as over-expression, normal expression and under-expression, respectively. Based on the median expression level of each gene, patients were divided into two groups: low and high, for each gene of interest. Then patients’ demographic data, hematological parameters, as well as cytogenetic abnormalities and mutations were compared between these groups. All data analyses were performed using SPSS Statistics 16.0 and Graph Pad Prism 8.4.2 software. P-values less than 0.05 were considered statistically significant. 

**Table1:** Primer sequences used in real-time PCR

**Primer**		**Sequence 5' → 3'**	**Amplicon size (b.p.)**
**BECLIN1**	F1	5′-CAA GAT CCT GGA CCG TGT CA-3′	191 bp
	R1	5′-TGG CAC TTT CTG TGG ACA TCA-3′	
**LC3**	F1	5′-ATG CCG TCG GAC AAG ACC TT-3′	360 bp
	R1	5′-TTA CAC TGA CAA TTT CAT CCC G-3′	
**GAPDH**	F1	5′-TGC ACC ACC AAC TGC TTA-3′	87 bp
	R1	5′-GAG GGC ATG GAC TGT GGT CAT-3′	

## Results

In this study, 61 patients including 24 AML, 20 ALL and 17 CML were enrolled. The demographic data, including age and sex for each group is demonstrated in tables 2-4. The control group was composed of 9 males and 9 females with the mean age of 37.44, ranging between 10 and 73 years. 

**Acute Myeloid Leukemia (AML): ** 24 AML patients included in this study. The mean age of patients is 45.45 years (±15.45), of which 13 (54%) were males. While the expression level of LC3 was significantly higher (2.25, P = 0.03), BECLIN1 expression was lower compared to healthy controls (0.6, p-<0.001). Most patients (mainly M3) under-expressed both BECLIN1 and LC3 genes (80% and 54%, respectively). There was no correlation between LC3 and BECLIN1 in AML patients (r: - 0.101, P= 0.653). The LC3^ high^ group indicated a significantly lower lymphocyte count than LC3^low^ (P= 0.023). As shown in table 2, patients in the BECLIN1^Low^ group showed significantly higher MPV levels than the BECLIN1^high^ group (P= 0.044). There was no significant relationship between the expression level of these genes and cytogenetic abnormalities and gene mutations.

**Acute Lymphoblastic Leukemia (ALL): ** Our study comprised 20 ALL patients of whom 70% were males. The mean age of patients was 13.6 (12.49). Although BECLIN1 expression was significantly lower (0.28, p<0.001), LC3 expression was not significantly different compared to the healthy controls. (1.16, P=0.184). While 15 (75%) of all patients under-expressed BECLIN1, 7 (35%) of them over-expressed LC3 gene. There was no correlation between LC3 and BECLIN1 in ALL patients (r: - 0.036, P=0.889). The LC3^high^ group showed a significantly lower HCT than the LC3^low^ group (P=0.017). There was no significant relationship between the expression of any of these genes and cytogenetic abnormalities and gene mutations (table 3).

**Chronic Myeloid Leukemia (CML):** Seventeen CML patients were enrolled in this study, of which 10 (58.8% were males with mean of age 39.23 (±14.78). According to our findings, 7 (41%) and 14(82%) of patients under-expressed LC3 and BECLIN1 genes, respectively. The mean expression of BECLIN1 was significantly lower than the control group (0.23, P= <0.001). However, the LC3 expression was not significantly different (1.66, P=0.075). Also, there was no correlation between LC3 and BECLIN1 in CML patients (r: -0.082, P=0.76). There was no significant association between the expression of any of these genes and hematological parameters and cytogenetic abnormality (table 4). 

**Table 1:** Laboratory finding and cytogenetic abnormalities of AML patients based on BECLIN1 and LC3 expression level

**Characteristics AML**		**Becline1 ** ^high^	**Becline1** ^ low^	**P-value**	**LC3 ** ^high^	**LC3 ** ^low^	**P-value**
**gender (male/ female), N=24**		6/6	7/5	1	8/4	5/7	0.414
**Age, mean, N= 24**		48.17 (14.44)	42.75 (16.58)	0.554	46 (18.29)	44.92(12.81)	0.092
**MCV, mean (S.D.), N=** ** 24**		90.73 (7.8)	91.6 (6.03)	0.439	92.21 (6.99)	90.11 (6.81)	0.688
**MCH , mean (S.D.), N=** ** 24**		29.8 (2.74)	31.15 (3.16)	0.884	31.13 (3.41)	29.83 (2.44)	0.41
**RBC, mean (SD), N= ** **24**		3.23 (1.58)	2.85 (0.88)	0.074	3.24 (1.54)	2.85 (0.95)	0.932
HGB*, mean (SD), N= ** 24**		9.45 (4.26)	8.8 (2.64)	0.328	9.9 (4.33)	8.35 (2.29)	1
HCT*, mean (SD), N= **24**		28.41 (11.51)	26.01 (7.89)	0.441	29.22 (11.9)	25.2 (6.77)	0.799
Platelet*, mean (SD), N= **24**		113.91 (103.54)	110.08 (94.93)	1	118.16 (92.65)	105.83 (105.23)	0.671
**Neut Absolute, mean (SD), N= 6**		55.1 (6)	57.83(16.1)	0.09	57.84 (11.68)	49.6	0.667
**Lymphocytes, mean (SD), N= 13**		28.58 (17.27)	26.55 (16.85)	0.735	25.21 (12.9)	30.21 (20.14)	0.023
RDW-CV*, mean (SD), N=24		17.1 (2.47)	20.32 (14.35)	0.1	21.31 (14.11)	16.11 (1.97)	0.092
WBC*, mean (SD), N= 24		36.4 (36.62)	50.43 (55.51)	0.932	38.39 (47.8)	48.45 (46.8)	0.266
**PDW, mean (SD), N=** ** 18**		12.37 (2.14)	11.93 (2.76)	0.363	12 (2.28)	12.31 (2.66)	0.254
**MPV, mean (SD), N= ** **17**		8.96 (0.59)	9.53 (1.28)	0.044	9.44 (1.09)	9.06 (0.98)	0.963
**AML with t(8;21)**	Yes	10 (92%)	11(83%)	1	11(83%)	10 (92%)	1
	No	2 (8%)	1 (17%)		1 (17%)	2 (8%)	
**AML with t (15;17)**	Yes	2 (17%)	5 (42%)	0.371	3 (25%)	4 (33.3%)	1
	No	10 (83%)	7 (58%)		9 (75%)	8 (66.7%)	
**FLT3-ITD mutation**	Yes	4 (33%)	2 (17%)	0.64	3 (25%)	3 (25%)	0.64
	No	8 (68%)	10 (83%)		9 (75%)	9 (75%)	
**NPM1 mutation**	Yes	3 (25%)	3 (25%)	1	2 (33%)	4 (17%)	0.640
	No	9 (75%)	9 (75%)		8 (67%)	10 (83%)	

Table2. Data are mean (SD) or n/N (%), where N is the number of patients with available data. P values were calculated by non-parametric tests (were indicated with *) and parametric tests based on the distribution of the LC3 and BECLIN1 expressions. Abbreviations: RBC: red blood cells; HCT: hematocrit; MCV: mean corpuscular volume; MCH: mean corpuscular hemoglobin; MCHC: mean corpuscular hemoglobin concentration; PLT: platelets; WBC: white blood cells; RDW-CV- Red Cell Distribution Width; PDW: Platelet Distribution Width

**Table 3:** Laboratory finding and cytogenetic abnormalities of ALL patients based on BECLIN1 and LC3 expression level

**ALL**		**Beline1 ** ^high^	**Beline1** ^ low^	**P-value**	**LC3 ** ^high^	**LC3 ** ^low^	**P-value**
**gender (male/ female), N=20**		5/5	9/1	0.051	7/3	7/3	1
**Age, mean, N= 20**		13.7 (13.02)	13.5 (12.64)	0.756	15.5 (13.4)	11.5 (11.8)	0.801
**MCV, mean (SD), N=** ** 20**		88.49 (6.64)	88.04 (6.95)	0.989	88.27 (5.63)	88.26 (7.8)	0.152
MCH* , mean (SD), N= **20**		27.21 (2.11)	27.18 (2.99)	0.755	26.69 (1.92)	27.7(3.02)	0.912
**RBC, mean (SD), N= ** **20**		5.65 (8.99)	2.86 (0.68)	0.056	5.55 (8.97)	2.96 (1.13)	0.08
HGB*, mean (SD), N= ** 20**		7.18 (2.8)	7.72 (1.78)	0.172	6.88 (1.62)	8.02 (2.79)	0.069
**HCT, mean (SD), N= ** **20**		24.57 (7.15)	25.06 (5.44)	0.546	24.16 (3)	25.47 (8.42)	0.017
Platelet *,mean (SD), N= **20**		129.5 (164.94)	103.5 (72.12)	0.971	150.8 (167.52)	82.2 (45.9)	0.529
**Neut Absolute ,mean (SD), N= 3**		58.35 (55.5)	17.1	1	18.1 (1.41)	97.6	0.667
Lymphocytes* , mean (SD), N= 18		60.26 (28.27)	78.02 (13.04)	0.113	76.2 (14.99)	60.32 (29.35)	0.122
RDW-CV*, **mean (SD),N=19**		15.75 (2.9)	17.1 (3.48)	0.549	15.54 (2.4)	17.29 (3.72)	0.315
WBC*, mean (SD), N= 20		69.42 (102.4)	44.29 (30.36)	0.739	53.6 (80.31)	60.11 (72.7)	0.481
PDW*, mean (SD), N= ** 12**		12.51 (3.05)	12.46 (3.6)	1	13.41 (4.29)	11.56 (1.34)	0.613
**MPV mean (SD), N= ** **12**		9.41 (1.2)	9.28 (1.0)	0.99	9.56 (1.38)	9.13 (0.67)	0.092
**ALL with t (1;19)**	Yes	0 (0%)	1 (10%)	0.305	1(10%)	0 (0%)	1
	No	10 (100%)	9 (90%)		9 (90%)	10 (100%)	
**ALL with t (12;21)**	Yes	3 (30%)	2 (20%)	0.606	3 (30%)	2 (20%)	1
	No	7 (70%)	8 (80%)		7 (70%)	8 (80%)	
**BCR-ABL P190**	Yes	2 (20%)	2 (20%)	1	3 (30%)	1(10%)	0.582
	No	8 (80%)	8 (80%)		7 (70%)	9 (90%)	

Table3. Data are mean (SD) or n/N (%), where N is the number of patients with available data. P-values were calculated by non-parametric tests (were indicated with *) and parametric tests based on the distribution of the LC3 and BECLIN1 expressions. Abbreviations: RBC: red blood cells; HCT: hematocrit; MCV: mean corpuscular volume; MCH: mean corpuscular hemoglobin; MCHC: mean corpuscular hemoglobin concentration; PLT: platelets; WBC: white blood cells; RDW-CV- Red Cell Distribution Width; PDW: Platelet Distribution Width.

**Table 4:** Laboratory finding and cytogenetic abnormality of CML patients based on BECLIN1 and LC3 expression level

**CML**		**Beline1 ** ^high^	**Beline1** ^ low^	**P-value**	**LC3 ** ^high^	**LC3 ** ^low^	**P-value**
**gender (male/ female), N=17**		5/4	5/3	0.614	4/4	6/3	0.608
**Age, mean, N= 17**		38.5 (10.56)	40.5 (19.57)	0.333	44.88 (16.41)	32.63 (11.63)	0.393
MCV* , mean (SD), N= ** 17**		86.05 (6.17)	85.01 (5.32)	0.574	83.48 (5.43)	86.32(5.85)	0.195
**MCH , mean (SD), N=** ** 17**		27.87 (3.06)	26.9 (2.67)	0.867	26.8 (2.56)	27.45 (3.12)	0.474
RBC*, mean (SD), N= **17**		4.33 (0.57)	4.16 (0.79)	0.878	4.57 (0.36)	3.99 (0.84)	0.234
**HGB , mean (SD), N=** ** 17**		12.12 (2.36)	11.13 (2.08)	0.622	12.28 (1.74)	10.93 (2.52)	0.241
HCT*, mean (SD), N= **17**		37.25 (5.49)	35.28 (6.4)	0.442	38.17 (3.84)	34.41 (7.12)	0.382
Platelet* , mean (SD), N= **17**		219.87 (49.97)	262.37 (167.32)	0.072	208.37 (55.74)	261.37 (167.71)	0.119
Neut Absolute*, mean (SD), N= 12		49.46 (22.96)	67.3 (12.51)	0.132	51.15 (23.74)	65.46 (15.23)	0.662
**Lymphocytes , mean (SD), N= 17**		31.83 (6.04)	32.32 (19.09)	0.183	32.3 (9.6)	34.86 (18.4)	0.365
RDW-CV*, **mean (SD), N=17**		15.28 (3.72)	15.33 (3.07)	0.866	15.48 (3.64)	15.88 (2.83)	0.667
WBC* , mean (SD), N= 16		7.07 (2.16)	16.77 (26.26)	0.798	6.53 (2.25)	16.82 (26.23)	0.645
**PDW , mean (SD), N=** ** 16**		12.35 (2.99)	11.28 (2.29)	0.755	11.8 (2.08)	11.57 (3.21)	0.465
MPV*, mean (SD), N= **16**		9.2 (0.68)	9.16 (1.32)	1	9.34 (1.2)	8.91 (0.94)	0.536
**BCR-ABL p210**	Yes	7 (87.5%)	6 (75%)	1	8 (100%)	5 (62.5%)	0.2
	No	1 (12.5%)	2 (25%)		0 (0%)	3 (37.5%)	

Table 4. Data are mean (SD) or n/N (%), where N is the number of patients with available data. P-values were calculated by non-parametric tests (were indicated with *) and parametric tests based on the distribution of the LC3 and BECLIN1 expressions. Abbreviations: RBC: red blood cells; HCT: hematocrit; MCV: mean corpuscular volume; MCH: mean corpuscular hemoglobin; MCHC: mean corpuscular hemoglobin concentration; PLT: platelets; WBC: white blood cells; RDW-CV- Red Cell Distribution Width; PDW: Platelet Distribution Width.

**Figure 1:**
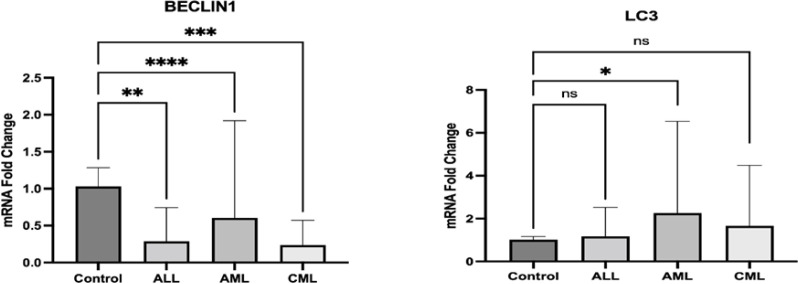
Comparison of mean expression of LC3 and Beclin1 genes in both patient and control groups in three types of leukemia

## Discussion

Different studies have shown the role of autophagy in cancer progression. However, its exact mechanisms in various stages of cancer progression need further investigations (20). Besides, fundamental differences between the pathogenesis of solid tumors and blood malignancies may suggest different autophagy role in leukemia pathogenesis. Due to the central role of BECLIN1 in the onset and progression of autophagy, an increasing number of studies focused on its expression status and prognostic value in a wide range of human cancers (13). 

The first observation which indicated an association between autophagy and cancer was the monoallelic deletion of BECLIN1 gene in breast, ovary, and prostate cancers. Although BECLIN1 was deleted in breast cancer cell lines, no mutations of BECLIN were observed (20, 21). In addition, the aberrant expression of BECLIN1 and LC3 has been shown in several solid tumors (22). According to previous reports, it appears that autophagy may play a role in both cancer progression and drug resistance. While decreased expression of autophagy genes may contribute to tumor growth in early stages, elevated expression may lead to drug resistance (23, 24).

The current study evaluated the expression of LC3 and BECLIN1 as two critical genes involved in the autophagy pathway in AML, ALL, and CML patients. AML is one of the heterogeneous hematologic malignancies characterized by different mutations and pathogenesis. Therefore, gene expression may show different patterns in AML subgroups. Our study indicated decreased BECLIN1 expression in AML patients compared to the control group (p <0.05). However, one of the limitations of the current study was the small sample size. Therefore, the relationship between gene expression and AML FAB subtype was not analyzed in our study. 

We observed a significantly lower expression of BECLIN1 in both AML and ALL patients compared to the control group, which was similar to the findings of Sara M. Radwan et al. They also reported reduced BECLIN1 expression in AML (30 cases) and ALL (25 cases) patients (25). However, a study on Chinese patients showed that BECLIN1 expression was higher in de-novo and refractory or relapse of acute leukemia patients than in the healthy group (26). 

The relationship between autophagy and genetic abnormalities in AML can be applied to autophagy modulation for the treatment of different AML subtypes to increase efficiency and overcome drug resistance (27). Moreover, a study by Yun Lian et al. showed that overexpression of BECLIN1 is associated with an unfavorable prognosis in AML patients. They suggested low BECLIN1 expression in AML patients with FLT mutation and monosomal chromosome 11 (28). Conversely, our study failed to explore the relationship between gene expression and patients' prognosis mainly due to a lack of access to the patients' outcome data. Besides, there was no correlation between BECLIN1 and mutations in our findings. Therefore, more studies are needed to elucidate the prognostic impacts of BECLIN1 expression in AML. Our study showed that LC3 expression was higher in AML patients compared to the healthy group (p<0.05). In contrast, the study of Mohamadimaram et al. (29) of AML patients found that LC3 gene expression was lower in AML patients. However, their study showed that LC3 expression was over expressed in 11.33% of AML patients. These differences may be related to small sample sizes and further studies with larger sample size needed to define changes in the expression of LC3 in AML patients. 

In a study of 50 patients with B-ALL, Hasanpour et al., found that most B-ALL patients had significant lower BECLIN1 expression compared to the healthy group (p<0.05). However, they found no significant correlation between different subgroups of B-ALL with BECLIN1 expression (30). Similarly, in our study, the expression of BECLIN1 was also lower in ALL patients. Additionally, Wang Z et al. suggested that Bortezomib, as a proteasome inhibitor, triggers autophagy in B-ALL cells by increasing the formation of the Beclin-1/PI3KC3 complex. They found that autophagy inhibitors can enhance the anti-ALL effects of Bortezomib (31). Hence, these findings may suggest potentials of autophagy for more investigation in ALL patients. 

Furthermore, we found decreased expression of BECLIN1 in CML patients. Similarly, some previous investigations suggested reduced BECLIN1 in leukemia cells (25, 32). In addition, higher Beclin1 expression was shown to have a tumor suppressor effect via autophagy activation (33, 34). In a previous study by Can G et al., they suggested that imatinib induces autophagy in CML cells by inducing increased expression of BECLIN1(35). BCR/ABL fusion protein is the most critical disease-causing factor of CML leukemia and hence BCR/ABL inhibitors are the most suitable therapies for CML treatment (36). Huang et al. found that the Beclin1 directly interacts with BCR/ABL and overexpression of BECLIN1 could be promising via BCR/ABL degradation in LCS-CML cells of patient's resistant to TKI(37). Furthermore, studies on leukemia patients resistant to treatment indicated overexpression of autophagy genes (38-40). Based on these findings, it seems that autophagy can play an important role in CML and further studies could shed more light on this issue. 

We assessed the expression of two crucial autophagy genes, including BECLIN1 and LC3 in patients with leukemia. The current study showed decreased expression of BECLIN1in AML, ALL, and CML patients as compared to the control group. In addition, the LC3 showed significantly decreased expression only in AML patients. On the contrary, some previous studies reported overexpression of these genes in therapeutic and relapsed patients. Since the molecular pathogenesis of leukemia subtypes can be different, the expression of Beclin 1 and LC3 genes in larger study groups should be analyzed to determine the relationship between gene expression levels and leukemia subtypes. In addition, the levels of gene expression in different stages of leukemia and its relationship with the type of treatment should be investigated.
